# Polyploidy but Not Range Size Is Associated With Seed and Seedling Traits That Affect Performance of *Pomaderris* Species

**DOI:** 10.3389/fpls.2021.779651

**Published:** 2022-01-13

**Authors:** Jason C. S. Chan, Mark K. J. Ooi, Lydia K. Guja

**Affiliations:** ^1^Centre for Ecosystem Science, School of Biological Earth and Environmental Sciences, University of New South Wales, Sydney, NSW, Australia; ^2^Centre for Australian National Biodiversity Research, a joint venture between Parks Australia and CSIRO, Canberra, ACT, Australia; ^3^National Seed Bank, Australian National Botanic Gardens, Parks Australia, Canberra, ACT, Australia

**Keywords:** functional trait, early life history, ontogeny, dormancy, germination, rarity

## Abstract

Ploidy and species range size or threat status have been linked to variation in phenotypic and phenological seed and seedling traits, including seed size, germination rate (speed) and seedling stature. There is surprisingly little known about the ecological outcomes of relationships between ploidy, key plant traits and the drivers of range size. Here we determined whether ploidy and range size in *Pomaderris*, a genus of shrubs that includes many threatened species, are associated with variation in seed and seedling traits that might limit the regeneration performance of obligate seeders in fire-prone systems. We experimentally quantified seed dormancy and germination processes using fire-related heat treatments and evaluated seedling performance under drought stress. We also examined the association of seed size with other seed and seedling traits. Polyploids had bigger seeds, a faster germination rate and larger and taller seedlings than diploids. There was a lack of any clear relationship between range size and seed or seedling traits. The ploidy effects observed for many traits are likely to be indirect and associated with the underlying seed size differences. These findings indicate that there is a higher potential competitive advantage in polyploid than diploid *Pomaderris* during regeneration, a critical stage in the post-fire environment. This insight to the regeneration phase may need to be considered when planning and prioritising management of threatened species.

## Introduction

Polyploidy, the condition of having more than two genome copies, is widespread in plants and thought to be a key evolutionary mechanism in the diversification of flowering plants (Hoya et al., 2007; Madlung, 2013). Polyploidy can have significant phenotypic consequences and is therefore likely to influence ecological performance (Ramsey and Ramsey, 2014). Polyploids are thought to have advantages over diploids that allow them to persist in challenging environments (Soltis and Soltis, 2000; Madlung, 2013; Ramsey and Ramsey, 2014). They can be more resilient or competitive under stress than diploids (Godfree et al., 2017; Stevens et al., 2020) and have an increased chance of becoming invasive (Soltis and Soltis, 2000; Hull-Sanders et al., 2009; Pandit et al., 2011; Thébault et al., 2011; Te Beest et al., 2012). In the few studies where the association between rarity or conservation status and ploidy levels has been investigated, diploids and species with a lower number of chromosomes had a higher chance of being threatened than polyploid species and species with a higher number of chromosomes (Pandit, 2006; Pandit et al., 2011). A community-level analysis of a fragmented landscape has shown that diploid plant populations have a much higher chance of facing local extinction than polyploid plant populations (Plue et al., 2018). Overall, polyploidy may affect a species’ relative evolutionary capacity, competitive dominance and conservation status.

An understanding of traits that limit performance of threatened or narrow endemic species can help scientists, natural resource managers and policy makers to set priorities for species conservation and population management (Schwartz, 1993; Lavergne et al., 2004; Pandit, 2006). Here we define species listed under conservation legislation as threatened and with a limited geographical distribution of less than 200 km^2^ [measured as Area of Occupancy (AOO), IUCN, 2012] as ‘narrow range’. We refer to any species with AOO greater than 200 km^2^ as ‘widespread’. Variation in species ranges is often attributed to the ability of species to tolerate climatic or other environmental conditions (Slatyer et al., 2013; Sonkoly et al., 2017). Comparative studies of threatened/narrow range and widespread species can identify limiting plant traits (e.g., Bevill and Louda, 1999; Burne et al., 2003; Lavergne et al., 2004) across life history stages. Seed and seedling stages are critical to understand because they are under particularly strong selective pressure in disturbance-driven ecosystems where species depend on recruitment from the seed bank for long-term persistence (Keith, 1996; Auld et al., 2000; Ooi et al., 2007; Saatkamp et al., 2019). For example, for fire-killed species in fire-prone regions, the soil seed bank is required for regeneration. Fire-related heat cues often alleviate seed dormancy and promote germination from the seed bank, with dormancy regulating the timing to coincide with a suitable post-fire environment (Keith, 1996; Ooi et al., 2014). While ploidy and range size have been studied in some fire-prone environments or species, there has not been a focus on their role in post-fire regeneration. Numerous phenotypic and phenological seed and seedling traits have been linked with endangered status or narrow range size in comparative studies. For example, endangered/narrow endemic species can have lower seed production (Pavlik et al., 1993; Walck et al., 2001; Lavergne et al., 2004), smaller seed size, slower germination, smaller stature and are less able to form a soil seed bank than more widespread species (Osunkoya and Swanborough, 2001; Walck et al., 2001; Burne et al., 2003; Lavergne et al., 2004; Mattana et al., 2010). Overall, endangered status or narrow range size appear strongly associated with several traits that limit plant performance or regeneration success.

There is still surprisingly little data on the ecological outcomes of relationships between ploidy and key plant traits (Plue et al., 2018), and studies rarely investigate multiple life history stages. Perhaps the most well-supported relationship is that genome multiplication in polyploids is associated with phenotypic traits, such as larger flowers and heavier seeds than diploids (Maceira et al., 1993; Bretagnolle et al., 1995; Hoya et al., 2007; Eliášová and Münzbergová, 2014; Godfree et al., 2017; Astuti et al., 2020; Stevens et al., 2020), which is often attributed to larger cell size. Whether ploidy related seed size variation results in the same well-known ecological benefits of larger seed size (Leishman et al., 1995, 2000; Moles and Westoby, 2004b), or if other factors offset these advantages, largely remains unknown. Heavier seeds in polyploids could enhance fitness as heavy seeds generally produce larger seedlings with a higher chance of survival in competitive environments (Bretagnolle et al., 1995; Liancourt et al., 2009). Another seed trait associated with ploidy is the level of dormancy within a seed lot. A study of meadow fescues in mountain ecosystems showed that tetraploid seeds were dormant whereas diploid seeds were not (Tyler et al., 1978). A study by Hacker (1988) on the African grass species *Digitaria milanjiana* across multiple ploidy levels also suggested a greater proportion of dormancy in populations with higher ploidy levels. While maternal environment may have also influenced the results in previous studies, Stevens et al. (2020) showed that even under common garden conditions, tetraploid plants of *Themeda triandra* (Poaceae) produced more dormant seeds than diploid plants. These examples indicate that ploidy differences within-species may be associated with significant variation in seed dormancy among populations, something that may also influence variation among species. While there are clear relationships between seed size and the speed of germination (rate; Norden et al., 2009) or seedling growth rate (Swanborough and Westoby, 1996), where smaller seeds germinate faster and have a faster seedling relative growth rate (RGR) than larger seeds, there are variable relationships between ploidy and seed germination or seedling growth rates. Polyploidy can be positively related to seed germination rate (Hoya et al., 2007; Astuti et al., 2020) and seedling growth rate (Levin, 1983; von Well and Fossey, 1998). On the other hand, polyploidy can be negatively related to seed germination rate (Levin, 1983) and seedling growth rate (Swanborough and Westoby, 1996). Further investigations of ploidy and growth rates, particularly for seedling traits, would provide critical information on regeneration and establishment potential.

Ecologically, polyploidy is thought to increase a plant’s ability to adapt to new environments and enhance stress tolerance, allowing polyploids to be distributed more widely than their diploid relatives (Levin, 1983; Soltis and Soltis, 2000; Čertner et al., 2019). Physiologically, the larger genome size in polyploids increases their hydraulic conductivity by enlarging xylem conduit size and stomata, potentially increasing a polyploid’s water retaining capacity and drought tolerance (Maherali et al., 2009). However, there may also be a risk of polyploids being more sensitive to water stress due to an increase in vulnerability to cavitation, resulting from increased xylem conduit size. Many polyploid species possess anatomical features that help reduce water loss, including fewer stomata per unit area and thicker epidermis (Levin, 1983; Li et al., 1996). There is a strong association between tolerance of water stress and survival, as well as numerous traits, such as reduction in growth rate and height, total biomass production (Pérez-Harguindeguy et al., 2016), relative amount of investment in root biomass (Comas et al., 2013; Larson and Funk, 2016) or trade-offs, such as smaller stature in exchange for higher seed production (Eliášová and Münzbergová, 2017). Experimentally, polyploids have been shown to have greater water uptake than diploids (Zaiats et al., 2020) and to be more resilient in drought conditions, for example, seed production was shown to be over four times higher in drought-stressed polyploids than diploids (Godfree et al., 2017).

Ploidy and range size may also impact regeneration in fire-prone ecosystems. A strong negative relationship exists between seed size and dormancy-breaking temperature thresholds, and there is a positive relationship between seed size and heat-related mortality (Hanley et al., 2003; Liyanage and Ooi, 2017a). Therefore, ploidy-driven variation in seed size could influence both the dormancy-breaking temperature threshold and seed survival during fire events. Seed size is also important for emergence as a high-intensity fire can kill seeds at shallow depths (Bond et al., 1999) and larger seeds may have an advantage as they are more capable of germinating successfully from deeper within the soil (Liyanage and Ooi, 2017a). Favourable conditions for seedling establishment tend to be short-lived in fire-prone systems and seeds with faster germination rates and faster seedling growth rates, leading to bigger seedlings at any given time, can have an advantage in capturing the increased availability of resources, such as light, space and nutrients (Moles and Westoby, 2004a; Denham et al., 2011). Factors, such as ploidy and range size, that can be related to traits during early life history stages may have important, perhaps interacting, impacts on regeneration and performance in fire-prone systems.

In this study we asked whether there are detectable effects of ploidy and/or range size on traits associated with seed dormancy, germination and establishment, that may affect performance during early life history in a genus of shrubs from fire-prone systems. We hypothesised that:

i. Driven by trait variation, ploidy and range size would interact to affect seed dormancy, germination and seedling growth.
ii. Polyploid or widespread species would produce larger seeds, and therefore lower dormancy-breaking thresholds, a faster germination rate and larger seedlings than their diploid or narrow range counterparts.
iii. Polyploid or widespread species would display a higher drought tolerance than their diploid or narrow range counterparts.

## Materials and Methods

### Study Species

One of the most effective ways to investigate potentially limiting traits is by using phylogenetically similar species (Burne et al., 2003). *Pomaderris*, with a range of widespread and narrow range/threatened species (Coates and Kirkpatrick, 1999; Kellermann et al., 2005; Messina et al., 2010) and diploid and polyploid species (Chen et al., 2019), is well suited to such investigations. A total of 15 *Pomaderris* species were selected based on seed availability, ploidy levels and range size/conservation status (Table 1).

**Table 1:** List of *Pomaderris* species selected for this study, including information on their State (NSW) threat level, range size (area of occupancy (AOO)), ploidy, genome size, habitat and fire response.

*Pomaderris* species	Categorisation	NSW threat status	Area of occupancy (km^2^)^#^	Inferred ploidy level^*^	Genome size (2C pg. ± SE)^*^	Habitat range	Fire response	References
*P. adnata*	*Narrow range, Diploid*	Endangered	8^1^	Diploid	0.927 (±0.004)	DSF, WSF	Seeder	Liyanage and Ooi, 2017a; NSW OEH, 2018
*P. andromedifolia*	*Widespread, Polyploid*	Not listed	328	Triploid	1.547 (±0.004)	DSF, heathland	Seeder	NSW OEH, 2014
*P. bodalla*	*Narrow range, Diploid*	Vulnerable	80	Diploid	0.966 (±0.004)	DSF, riparian	Seeder	Le Breton, 2016; NSW OEH, 2018
*P. brunnea*	*Narrow range, Diploid*	Endangered	160	Diploid	0.945 (±0.005)	DSF, WSF, GW, riparian	Seeder	Sutter, 2011; NSW OEH, 2014; NSW OEH, 2018
*P. cotoneaster*	*Narrow range, Polyploid*	Endangered	136	Tetraploid	1.862 (±0.005)	DSF, WSF, GW, riparian	Seeder	NSW OEH, 2014; NSW OEH, 2018
*P. elachophylla*	*Widespread^^^, Polyploid*	Endangered^^^	476	Triploid	1.393 (±0.007)	DSF, GW, riparian	Seeder	NSW OEH, 2014; NSW OEH, 2018
*P. eriocephala*	*Widespread, Polyploid*	Not listed	624	Triploid	Approximately 1.3	DSF, GW	Seeder/ Resprouter	NSW OEH, 2014
*P. intermedia*	*Widespread, Polyploid*	Not listed	1,036	Triploid	1.636 (±0.002)	DSF, forested wetlands	Seeder	NSW OEH, 2014
*P. lanigera*	*Widespread, Diploid*	Notlisted	1,712	Diploid	Approximately 1.0	DSF, forested wetlands	Seeder	NSW OEH, 2014
*P. ligustrina* subsp*. ligustrina*	*Widespread, Diploid*	Not listed	284	Diploid	0.998 (±0.003)	Riparian	Unknown	Walsh, 2017
*P. pallida*	*Narrow range, Polyploid*	Vulnerable	160	Triploid	1.394 (±0.006)	Riparian, shrub communities surrounded by woodland	Unknown	NSW OEH, 2018
*P. prunifolia*	*Widespread, Polyploid*	Not listed	292	Triploid	Approximately 1.4	WSF	Seeder	NSW OEH, 2014
*P. reperta*	*Narrow range, Polyploid*	Critically endangered	10^2^	Triploid	1.497 (±0.005)	DSF, WSF	Seeder	NSW OEH, 2018
*P. velutina*	*Widespread, Diploid*	Not listed	260	Diploid	0.894 (±0.001)	Riparian	Unknown	PlantNet, 2018
*P. walshii*	*Narrow range, Polyploid*	Critically endangered	8^2^	Tetraploid	1.993 (±0.002)	DSF, riparian	Seeder	Liyanage and Ooi, 2017a; NSW OEH, 2018

# Area of occupancy estimates from Gallagher et al. (2021), except ^1^Le Breton et al. (2020) and ^2^Conservation Advice for the Australian Government’s Environment Protection and Conservation Act 1999.

* Inferred ploidy levels and genome size estimates [2C value in pg (±standard error for triplicate measurements) or approximate estimate based on a single measurement from a seed sample] from Chen et al. (2019).

^ Although *P. elachophylla* is listed as endangered in NSW, its AOO is >200 km^2^ and it occurs in greater numbers in other states so was categorised as ‘wisdespread.’

Habitat range: DSF, dry sclerophyll forest; WSF, wet sclerophyll forest; GW, grassy woodland.

References: NSW OEH, NSW Office of Environment and Heritage.

*Pomaderris* (Rhamnaceae) consists of shrubs and small trees that occur in Australia’s fire-prone ecosystems (Kellermann et al., 2005). There are 68 *Pomaderris* species in Australia, most of which are endemic, occurring primarily across southern temperate regions (Kellermann et al., 2005). Most *Pomaderris* species are seeders that have soil seed bank storage (NSW Office of Environment and Heritage, 2014). *Pomaderris* seeds have physical dormancy (Turner et al., 2005; Haines et al., 2007; Ooi et al., 2014; Liyanage and Ooi, 2017a; Wood, 2020), which is alleviated by heat shock (for example fire-related heating of the soil; Liyanage and Ooi, 2017b; Le Breton et al., 2020). Of the 68 *Pomaderri*s in Australia, 17 are nationally listed as threatened (Department of the Environment and Energy, 2018), with further listings at state and territory level (Sutter, 2011; Le Breton, 2016). Many of the species (45 out of 68) occur in NSW, with 16 currently listed as threatened in NSW (Millott and McDougall, 2005; NSW Office of Environment and Heritage, 2018).

We focused the study on *Pomaderris* collections from the state of NSW and therefore used the threat status assigned at State level. The majority of study species have the centre or much of their distribution, occurring within the State of NSW. To assign species as narrow range or widespread we considered the estimated AOO across Australia (Table 1). AOO is defined as the smallest area occupied by a species, excluding vagrants (IUCN, 2012). Estimates of AOO were obtained from the literature (Le Breton et al., 2020; Gallagher et al., 2021). Species that have been assigned a threat level (critically endangered, endangered, or vulnerable) in NSW and had a relatively small AOO were categorised as ‘narrow range.’ Species that have not been assigned a threat-level or had a relatively larger AOO (>200 km^2^) were categorised as ‘widespread’ (Table 1).

Flow cytometry is a rapid and reliable method of identifying polyploid complexes among close relatives due to the relationship between ploidy and nuclear DNA content. The genome size (2C pg) of mature plants of each *Pomaderris* species, sometimes the mother plants for the seed lot, was measured in triplicate using flow cytometry by Chen et al. (2019; Table 1). In short, *Pomaderris* leaf samples and a standard were processed in buffer solutions, stained and loaded into a flow cytometer until at least 5,000 events were counted in total per sample. In some cases, if a leaf sample was not available a seed sample was assessed to obtain an approximate estimate of genome size from seed samples and was not replicated (Table 1). Chromosome counts were also conducted by Chen et al. (2019) for selected species to inform interpretation of flow cytometry results. For detailed flow cytometry and chromosome count methodology, see Chen et al. (2019). Whether the study species are autopolyploids or allopolyploids could not be determined and is beyond the scope of these studies, and our investigations were therefore based on broadly comparing diploids with polyploids. Species that were diploid were categorised as ‘diploid’ and species of higher ploidy (triploid or tetraploid) were categorised as ‘polyploid’ (Table 1).

### Seed Collections

*Pomaderris* seeds were obtained from the seed bank facilities at the Australian Botanic Garden, Mount Annan and Australian National Botanic Gardens, Canberra. All seeds were collected from wild populations by botanic garden’s staff and Dr. Mark Ooi under relevant permits. For most seed lots assessed in this study, nylon mesh bags were placed over the seeding branches of multiple mother plants per population to ensure the collection of mature seeds. Seeds from mothers within a population were combined into a population sample. Once collected, a vacuum separator was used to separate seeds from chaff. Seeds were then stored in dry dark conditions (15°C and 15% RH) or in dark, dried and frozen conditions (−20°C) at the seed bank facilities prior to use. Such storage conditions have previously been shown to have minimal effects on seed dormancy or viability characteristics of many species with physically dormant seeds, particularly those that require heat shock to overcome dormancy, such as *Pomaderris* (Merritt et al., 2003; Vasques et al., 2014). We used 400–600 seeds for each of the 15 species in this study, depending on seed availability. Not all species were used for all measured seed and seedling traits due to seed availability (See Supplementary Appendix S1 for the species used in each measured trait).

### Seed Traits

Twenty seeds were randomly selected from the seed lots of each species and the individual seed mass measured using a fine-scale balance (A&D GR-202, Japan) with mean mass calculated and expressed in milligrams (mg). Initial viability of seed lots was estimated from seed fill, using three replicates of 20 seeds imaged with an X-ray imaging system (Faxitron MX-20 Cabinet X-ray System, United States).

To identify dormancy-breaking requirements and enable calculation of germination rate (speed), heat treatments were used, and the response was assessed by germination trials. Dormancy is usually overcome by a high temperature heat shock, so seeds were subjected to a range of dry heat treatments simulating temperatures the seed bank would be exposed to in the upper soil profile during fire. Seeds were exposed to four levels of dry heat for 10 min in an oven (LABEC Model ICC36-HT, Australia): 60, 80, 100 and 120°C. The 120°C treatment was chosen as the upper limit as it had been found to be close to the limit for seed mortality in physically dormant seeds (Ooi et al., 2014). Four replicates of 20–25 seeds were used per species for each temperature and for the unheated treatment control (except *P. intermedia* where only three replicates for heat and control treatments were used due to low seed availability), one replicate at a time, to minimise pseudoreplication within each treatment level (Morrison and Morris, 2000). After heat shock treatments to overcome dormancy, seeds were allowed to cool and then put into 90 mm petri dishes on moistened filter paper. Dishes were wrapped with parafilm to reduce evaporation and transferred to an incubator (LABEC Model ICC36-HT, Australia) set at a temperature cycle of 25/18°C on 12 h/12 h light/dark cycle to mimic the approximate natural temperature variation during summer, the fire season in NSW (Gill, 1975). Dishes were randomly placed within the incubator and rotated once every week. Seeds were checked two times a week and germination was scored upon radicle emergence (radicle >1.5 mm). Germinated seeds were immediately removed from the dish for planting, and mouldy seeds were recorded as inviable and removed from the dish to prevent contamination. Distilled water was added to the dishes when necessary to maintain moisture levels. The germination trial for each species was terminated when the cumulative germination reached a plateau.

To assess germination speed, we used data from the temperature with maximum germination for each species. Using the drm function in the *drc* package in R 3.5.1 (Ritz et al., 2015; R Development Core Team, 2018), a log-logistic dose–response curve was fitted, using the number of seeds germinated against the day at which they germinated. Curves were fitted for each species and the time to reach 50% (T_50_) was calculated by inverse regression.

To compare the minimum dormancy-breaking temperature thresholds, the lowest level of dry heat that produced at least 20% seed germination (G_20_) for each replicate was recorded. The recorded temperature was then averaged across all replicates within each species to generate the mean G_20_ for each species. As upper threshold or optimum dormancy-breaking temperatures are likely to be very similar (Ooi et al., 2014) we instead used the G_20_ index to estimate the lower-bound variation in dormancy alleviation.

The percentage of seed mortality was calculated at 120°C, the highest level of dry heat treatment and representative of a high-intensity fire. To assess seed mortality after heat treatment, cut tests were performed on ungerminated seeds from the 120°C heat-treated seeds, after the termination of the germination trial. Seeds with white, firm endosperm were scored as viable and black, yellow, brown and spongy seeds were scored as inviable (Ooi et al. 2004).

### Seedling Traits and Drought Tolerance

A glasshouse experiment was conducted to compare seedling performance under watered and drought conditions. For each species, 40 to 50 germinated seeds were randomly selected from the germination trial. Each germinated seed was transplanted into a 5 cm × 5 cm × 12 cm pot filled with potting mix, with geotextile placed at the bottom of each pot to hold the soil while allowing sufficient drainage. One cubic metre of potting mix contained one part of Coir peat and two parts of sand with fertiliser (Dolomite: 435 g; Iron Sulphate: 500 g; Micromax: 365 g; Osmocote: 1,500 g). All pots were placed in a random order and rotated once every 3 weeks to capture the potential variation in sunlight exposure inside the glasshouse. All seedlings were grown for 3 weeks with daily watering to field capacity (‘settlement phase’) to reduce the chance of transplantation mortality immediately upon transfer from the petri dish (Maherali et al., 2009). Seedlings were watered for 2 min at 8 am and 1 min at 4 pm in each daily watering. Between 14 and 47 seedlings emerged and survived the three-week settlement phase per species, leaving most species with at least 30 seedlings for the experiment (except *P. velutina*, 20 seedlings and *P. pallida*, 14 seedlings).

After the three-week settlement phase, drought treatments were applied to half of the plants. The drought treatment aimed to stress plants without causing mass mortality and included both a reduction in how often pots were watered and two extended periods (7–10 days at weeks 3 and 7) of no watering. The control group was watered daily for 6 weeks while the treatment group was watered twice a week (‘drought treatment’) in week 1, 2, 4 and 5. The overall water loss was calculated over a 9-day duration of no watering (see Supplementary Appendix S2), with a mean change of 0.21 (*θ*_g_).

The temperature range was monitored by a temperature data logger (DS1921G-F5 thermochrons) throughout the experiment for the Control (14.1 ± 0.07°C) and Drought (17 ± 0.07°C) treatments.

To assess the effects of our drought experiment, we measured plant survival and other attributes at the commencement and at the end of the drought treatment. Stem length of individual seedling in both treatments was measured using digital calipers and expressed in millimetres (mm). RGR was then calculated based on stem length, as it is one of the most sensitive stress indicators for water deficits. Mean RGR of each species was calculated by averaging the RGR across all treatment replicates within each species. To calculate RGR for each plant, we used the following formula:


$$RGR=\left(\ln S_{2}-\ln S_{1}\right)/\left(T_{2}‐T_{1}\right)$$

where *S*_1_ and *S*_2_ were the stem length of the plants at *T*_1_ (first day of the treatment phase) and at *T*_2_ (final day of the treatment phase).

Dry biomass was also measured at the end of the growth experiment. Shoots and roots from a subsample of five plants per species from each treatment were collected within a week after the final measurement of stem length was taken. Both shoots and roots were washed by hand to remove the soil (Pérez-Harguindeguy et al., 2016), oven-dried at 60°C for 72 h and weighed to estimate total biomass production. The shoot:root ratio (SRR) was calculated for each plant to determine the distribution of dry plant biomass. Mean biomass and mean SRR for each species were calculated by averaging all replicates within each species.

### Statistical Analyses

All statistical analyses were conducted in R (R version 3.5.1; R Development Core Team, 2018) using the RStudio integrated development environment (RStudio version 1.1.453; RStudio Team, 2018).

Four seed traits (seed mass, T_50_, G_20_, mortality) and five seedling traits (RGR stem length, stem length, SRR, total biomass and survival) were assessed. The two main predictors, ploidy and range size, had two levels, diploid or polyploid and narrow range or widespread, respectively (Table 1). Additionally, for the third hypothesis, there was a third predictor (drought treatment) for the seedling traits. The effect of the predictors on continuous response data (seed mass, G_20_, RGR stem length, stem length, SRR and total biomass) was analysed using Linear Mixed-effects Models (LMM), except for T_50_, which was analysed using a linear model (only a single value per species). The effect of predictors on proportional mortality data was analysed using a Generalised Linear Mixed-effects Model (GLMM) using a binomial distribution with logit link function. These two sets of models were fitted with the *lme4* package (R Development Core Team, 2018), using species as a random factor. We were unable to directly account for phylogenetic relationships with our analyses because at the time of study a phylogeny was not available for the genus. We therefore accounted for relatedness by including species as a random factor in our analyses. A Generalised Linear Model (GLM) assuming a binomial distribution with logit link function was used to test the effect of predictors on seedling survival. We checked for any potential effects of seed age by assessing storage duration (months) against the germination parameters T_50_, G_20_ (linear models) and seed mortality (binomial model) and found no significant relationships (data not shown).

All response variable data were checked to ensure normality of residuals and homoscedasticity assumptions. T_50_ values underwent a Tukey ladder of Power transformation to fulfil the homoscedasticity assumption. G_20_ and stem length data were log-transformed to improve the normality of residuals and increase the homoscedasticity of variance. A single outlier replicate was removed from the seed mortality data set as it had three times greater mortality than other replicates, suggesting an experimental anomaly.

Model selection was utilised for each of the seed and seedling traits. For the four seed traits, the initial model contained the two main effects: ploidy and range size and their interaction. For the five seedling traits, the initial model contained three main effects: drought treatment, ploidy, range size and their interactions (interaction between ploidy and range size was excluded due to low seedling availability). All combinations of reduced models (five in total including the null model for the seed germination experiment, 23 in total including the null model for the glasshouse drought experiment) were then produced for each trait (response variable). The models were ranked with Akaike’s Information Criterion corrected for small sample sizes (AIC_c_) using the MuMin package (R Development Core Team, 2018). The model with the lowest AIC_c_ was chosen as the final model to capture all potential important terms. Where the null model had the lowest AIC_c_ and there was competing support (Δ AIC_c_ < 2), the next best model was selected. Values of *p* were obtained through likelihood ratio tests by comparing the final model against a reduced model excluding fixed effects, using the *anova* function in R.

To further examine the relationship between seed mass and each seed and seedling trait, Pearson’s correlation coefficients were calculated, or binomial models fitted (for proportion seed mortality and seedling survival), between log-transformed seed mass and each trait. Note that for seedling traits, data were combined from both watered and drought treatments. Tukey’s Ladder of Power transformation was used when necessary to improve the normality of residuals of traits.

## Results

**Figure 1:**
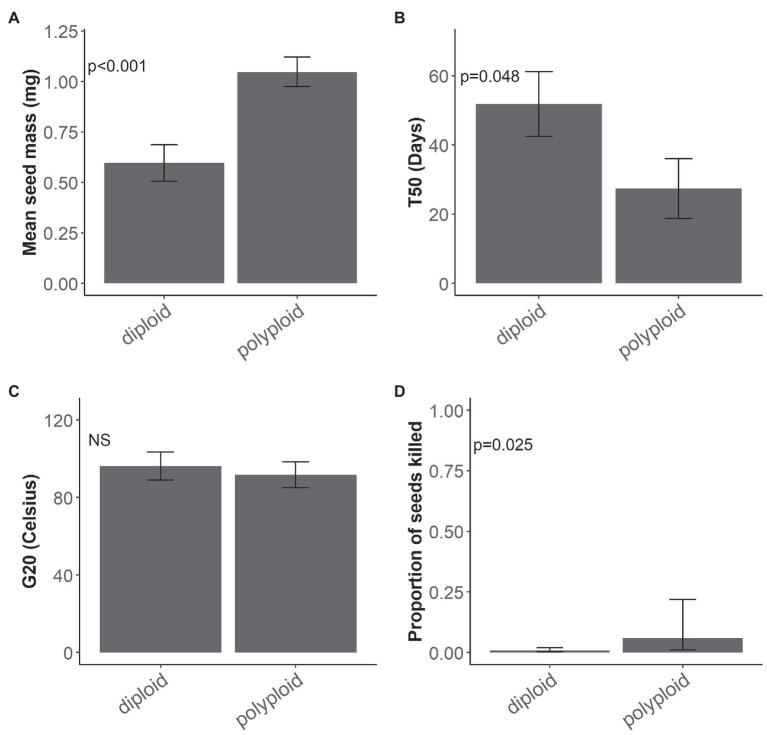
Ploidy and seed traits. Mean and standard error for **(A)** seed mass (mg; *n* = 20 per species), **(B)** germination speed T_50_ (days; *n* = 4 per species), **(C)** minimum dormancy-breaking temperature thresholds G_20_ (°C; *n* = 4 per species) and mean and confidence interval for **(D)** seed mortality (proportion of seeds killed) at 120°C treatment, of diploid and polyploid *Pomaderris* (*n* = 4 per species). NS indicates a non-significant value.

There was no interaction between ploidy and range size influencing the four traits measured in the seed germination experiment. Ploidy, often as the sole main factor, had the greatest effect on seed traits (Table 2; Supplementary Appendix S3) and it was a strong driver of the differences observed between traits at the seed stage. Polyploid seeds were significantly heavier than diploid seeds (*χ*^2^ = 11.555, d.f. = 1, *p* < 0.001; Figure 1A) and reached 50% germination (T_50_) significantly faster than diploid seeds (*χ*^2^ = 4.953, d.f. = 1, *p* = 0.048; Figure 1B). G_20_ was similar for polyploids (94°C) and diploids (96°C; *χ*^2^ = 0.2684, d.f. = 1, *p* = 0.604; Figure 1C). Although seed mortality overall was relatively low (<15%), significantly more polyploid than diploid seeds were killed after the 120°C heat treatment (*χ*^2^ = 4.972, d.f. = 1, *p* = 0.025; Figure 1D).

**Table 2:** Summary of results for all traits and factors investigated.

Life stage	Trait	Ploidy	Range size	Drought	Relationship with seed mass
Seed	Seed mass	^***^		NA	NA	
	T_50_	^*^		NA	−ve	
	G_20_			NA	−ve	
	Seed mortality	^*^		NA	+ve
Seedling	RGR (stem)		^*^	^*^	ns	
	Stem length				+ve	
	SRR				+ve	
	Biomass	^*^		^*^	+ve	
	Survival			^***^	ns

Shaded cells indicate that the factor was included in the best-fitting model identified by lowest AICc. Asterisks denote significance at less than 0.05 (^*^), 0.01 (^**^) or 0.001 (^***^) for the main factors: ploidy level, range size and drought treatment. Significant relationships with seed mass (mg) are denoted as positive (+ve), negative (−ve) or non-significant (ns). NA denotes factor not tested.

Ploidy also had an effect at the seedling stage, although for fewer variables (Table 2). The full model developed for each seedling trait included interaction terms between ploidy and treatment, range size and treatment, plus their main effects. Removing the interaction terms considerably improved the AIC_c_ scores across all traits. The main effects of either ploidy or treatment or both were included in most of the best-fitting models (Table 2; Supplementary Appendix S4).

**Figure 2:**
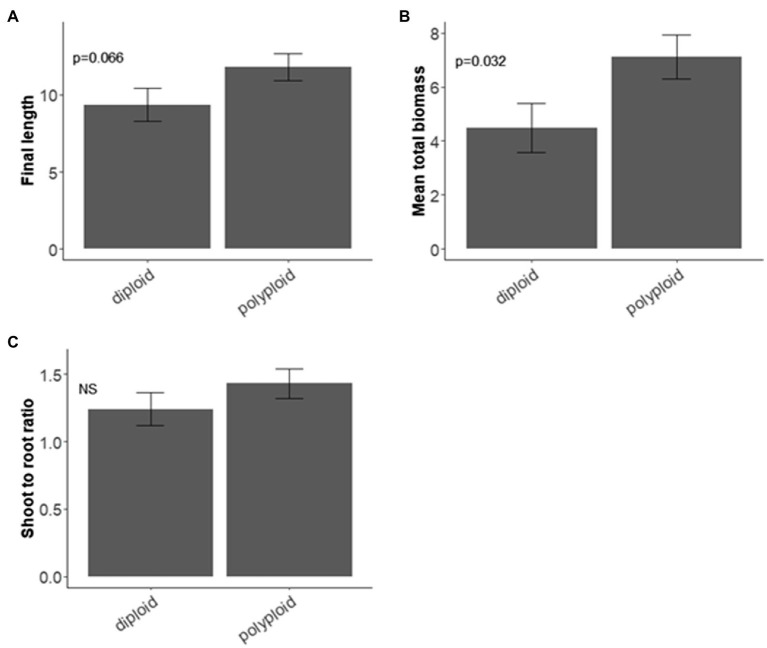
Ploidy and seedling traits of *Pomaderris*. Mean and standard error for **(A)** stem length (mm) at 6 weeks (*n* = 25 per species for each treatment), **(B)** biomass (mg; *n* = 5 per species for each treatment), **(C)** biomass shoot to root ratio (SRR; *n* = 5 per species for each treatment). NS indicates a non-significant value.

Polyploids had taller stems than diploids but this difference was not significant (*χ*^2^ = 3.373, d.f. = 1, *p* = 0.066; Figure 2A), regardless of drought treatment. The biomass of polyploids was significantly heavier than diploids (*χ*^2^ = 4.607, d.f. = 1, *p* = 0.032; Figure 2B). The shoot:root ratio did not differ significantly between diploids and polyploids (Figure 2C).

The drought treatment had a strong effect on seedling traits (Supplementary Appendix S4), indicating that it imposed stress; however, the interaction between ploidy level and drought was not in the best-fitting models for any of these traits. Not surprisingly, drought significantly reduced seedling survival compared to watered controls (*χ*^2^ = 28.672, d.f. = 1, *p* < 0.001) and significantly slowed the RGR (*χ*^2^ = 4.600, d.f. = 1, *p* = 0.032). For SRR, the best-fitting model included only drought treatment as the fixed factor, with smaller values under drought (although not significant; *χ*^2^ = 3.474, d.f. = 1, *p* = 0.062), suggesting greater partitioning of resources towards root growth under stressful conditions.

Range size did not appear to be a strong driver of responses for any of the traits measured, although seedlings had a significantly greater RGR than widespread species, as demonstrated by the significant association between range size and the RGR of stem length (*χ*^2^ = 5.818, d.f. = 1, *p* = 0.016). There was also a significant main effect of drought on RGR, with reduced rates under drought conditions (*χ*^2^ = 4.600, d.f. = 1, *p* = 0.032).

**Figure 3:**
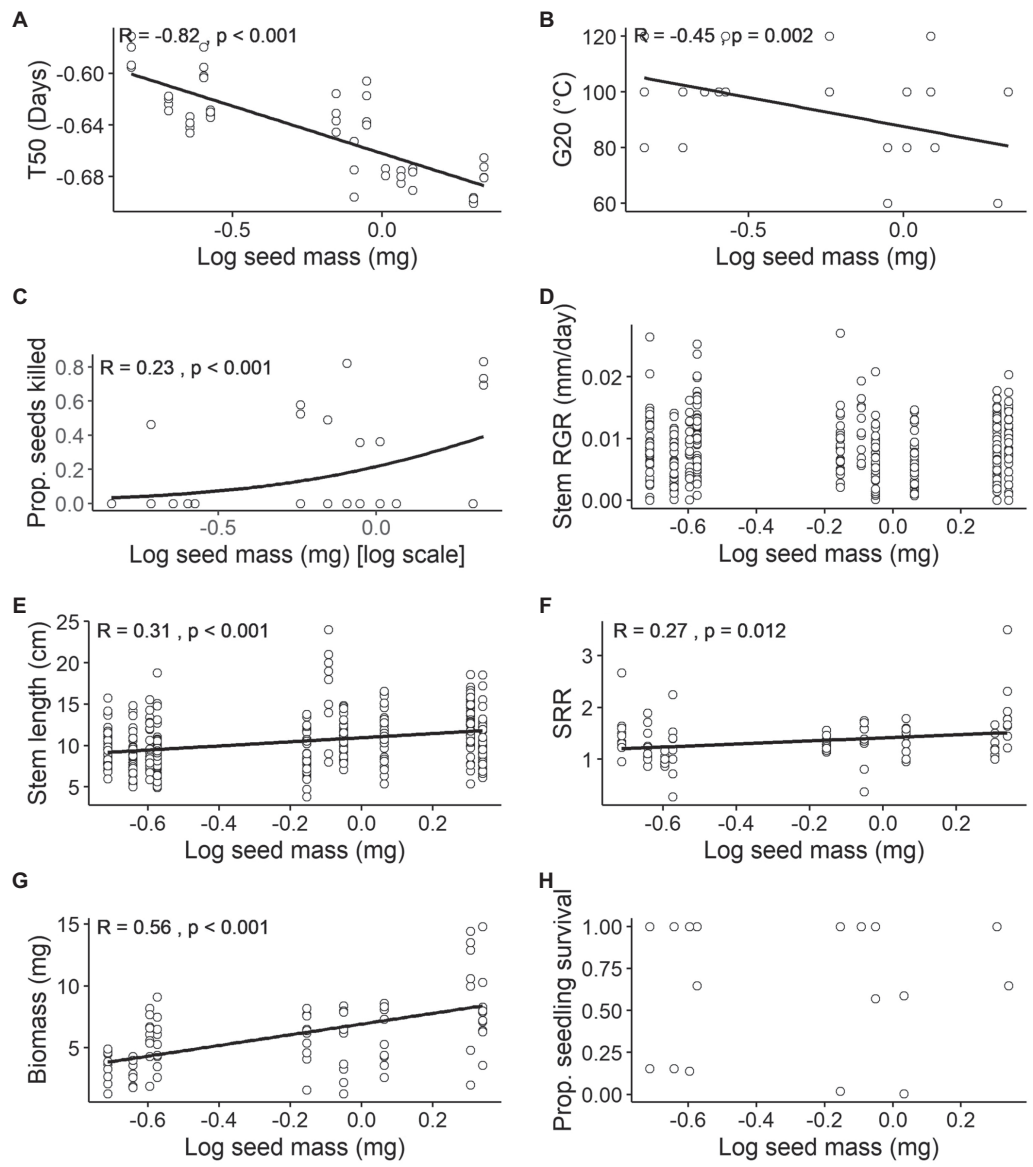
Relationships between mean seed mass (mg) [log scale] and seed and seedling traits. Sample size (*n*) varies within each trait. **(A)** Germination speed T_50_ (days; *n* = 4 per species), Tukey’s ladder transformation, **(B)** minimum dormancy-breaking temperature thresholds G_20_ (°C; *n* = 4 per species), **(C)** seed mortality (proportion of seeds killed) at 120°C treatment (*n* = 4 per species), **(D)** Relative growth rate (RGR) of stem length (mm/day; *n* = 25 per species for each treatment), **(E)** stem length (mm; *n* = 25 per species for each treatment), **(F)** shoot to root ratio (SRR; *n* = 5 per species for each treatment), **(G)** biomass (mg; *n* = 5 per species for each treatment) and **(H)** seedling survival (proportion of survived seedlings calculated based on (*n* = 25) sample size per species per treatment) on combined watered and drought treatment. All continuous variables were tested using Pearson’s correlation and binomial responses using Generalised Linear Models (GLMs). For significant relationships, the line of best fit has been plotted. No line denotes non-significant associations among traits.

Most seed traits displayed significant relationships with seed mass (Figures 3A–H). There was a strong negative linear relationship between T_50_ and seed mass (*r* = −0.82, *p* < 0.001; Figure 3A). G_20_ also exhibited a significant negative relationship with seed mass (*r* = −0.45, *p* = 0.002; Figure 3B). There was a positive relationship with seed mortality, showing a trend of higher mortality for heavier seeds at high temperatures (Binomial model, Pseudo *r*^2^ = 0.23, *p* < 0.001; Figure 3C).

For seedling traits, RGR and seedling survival did not show any significant correlation with seed mass (*r* = 0.008, *p* = 0.881; Psuedo *r*^2^ = 0.006, *p* = 0.452; Figures 3D,H), while stem length, SRR and biomass were all positively correlated with seed mass (stem length: *r* = 0.31, *p* < 0.001; SRR: *r* = 0.27, *p* = 0.012; biomass: *r* = 0.55, *p* < 0.001; Figures 3E–G). While the results indicate that larger seeds result in larger seedlings (Figure 3E) they also demonstrate that RGR was independent of seed size (Figure 3D).

## Discussion

Relatively few studies have investigated the ecological consequences of polyploidy (Ramsey and Ramsey, 2014; Plue et al., 2018). Our study has demonstrated that polyploids maintain characteristics in the regeneration stage (faster germination rate and larger and taller seedlings) that contribute to a potentially higher competitive advantage than diploids. Hence, polyploid seeds that survive the passage of fire might have a higher chance of recovering quickly after fire (or other disturbance) than diploids. The larger seed size of polyploid *Pomaderris* may be an underlying mechanism driving some of these key differences in regeneration traits, but seed size is not the only driver. Our investigations showed that range size and ploidy did not interact significantly and, in fact, ploidy alone was significant for most of the seed and seedling traits measured. Overall, these findings indicate that ploidy has a strong influence on performance and several key traits in the regeneration phase of *Pomaderris*, which may have ecological consequences for species persistence in fire-prone habitats.

Polyploid *Pomaderris* seeds were significantly heavier than diploid seeds, consistent with studies of other plant species and ecosystems (Maceira et al., 1993; Hoya et al., 2007; Eliášová and Münzbergová, 2014, 2017; Godfree et al., 2017; Stevens et al., 2020). This pattern is generally attributed to genome multiplication causing an increase in cell size which results in a larger seed size (Bretagnolle et al., 1995; Te Beest et al., 2012). Polyploid *Pomaderris* germinated significantly faster than diploids, adding to the growing body of evidence that demonstrates polyploid germination to be faster than diploid germination (Bretagnolle et al., 1995; Hoya et al., 2007; Eliášová and Münzbergová, 2014). The mechanisms underlying larger seed size and faster germination of polyploids are not yet well characterised. Potentially, the additional genome in polyploid seeds might result in a higher energy content, as well as a larger energy requirement and thus lead to more rapid food reserve mobilisation to support growth (von Well and Fossey, 1998). Ecologically, larger seeds and faster germination, regardless of whether they are effects of seed size or other polyploidy effects (Bretagnolle et al., 1995; Eliášová and Münzbergová, 2014), can be advantageous traits in the post-fire environment.

The high temperatures required to overcome physical dormancy in the relatively small-seeded *Pomaderris* species in this study (a G_20_ mean of approximately 95°C) is consistent with the concept that small-seeded species in fire-prone systems have higher dormancy-breaking temperature thresholds than large-seeded species (Hanley et al., 2003; Ooi et al., 2014; Liyanage and Ooi, 2017a). *Pomaderris* and other small-seeded species close to the soil surface are more likely to be exposed to higher temperatures during the passage of fire. Due to their higher temperature thresholds for dormancy alleviation, they emerge from closer to the soil surface and avoid germination from depths too deep for small seeds to emerge successfully (Ooi et al., 2014; Liyanage and Ooi, 2017a). The positive relationship we found between seed size and seed mortality at high temperatures is further evidence of smaller seeds from fire-prone systems being adapted to survive high temperatures in the soil. However, the dormancy-breaking temperature threshold (measured by our G_20_ index) did not differ significantly between ploidy levels. There was significantly higher mortality in polyploid than diploid species as well as a strong correlation with seed mass (smaller seeds had a higher G_20_ and suffered less seed mortality at 120°C).

The hypothesis that polyploid *Pomaderris* would have a higher drought tolerance than their diploid counterparts was not supported for any seedling traits assessed, a finding that differs from other studies investigating water stress response and ploidy (e.g., Li et al., 1996; Eliášová and Münzbergová, 2017). The drought treatment in our study significantly decreased survival, yet did not result in a stress-related advantage for polyploids. Drought tolerance may be reflected in a greater investment in root biomass for better water acquisition (Comas et al., 2013); however, there was no clear difference in the investment of resources between diploids and polyploids, and there was only a relatively weak relationship between SRR and seed mass (*r* = 0.27). Therefore, polyploid *Pomaderris* do not seem to be more stress tolerant or differ from diploids in their root investment under drought. Other studies have found that root investment under water stress is variable or species-specific and could be due to other mechanisms (Larson and Funk, 2016). The stress response of *Pomaderris* seedlings may differ for mature seedlings or adult plants during the plant life-cycle (Satyanti et al., 2021) and could be investigated in future.

The lack of any clear effect of range size on most of the performance traits we studied may be related to the multiple potential causes of narrow range size and threatened status. Most trait relationships with range size or threat have a highly context-dependent nature (Bevill and Louda, 1999; Murray et al., 2002). The one significant effect of range size found in our study, faster RGR for stem length, as well as a tendency for lower mortality in narrow range species, showed that narrow range species might not always perform worse than widespread species. However, outside of a controlled environment other external factors, such as competition, can affect species rarity (McIntyre, 1995). Disturbance dependent species in Australia are more likely to be threatened when there is an absence of appropriate disturbances and an increase in exotic plant competition, herbivory or other factors (McIntyre, 1995). Because of the broad range of potential factors driving range size and threatened status, future studies could consider ploidy and range size for a larger number of species and multiple potential drivers (e.g., analysing genome size, range size, niche breadth, latitude, and altitude) which may reveal other relationships (Pandit et al., 2014). The phylogenetic relationships among species may also influence results. At the time of this study, a comprehensive phylogeny of the genus was not available. The recent publication by Nge et al. (2021) of a *Pomaderris* phylogeny will provide opportunities to better account for relationships among species in future studies. Informal comparison of the ploidy results from Chen et al. (2019) and the recent phylogeny (Nge et al., 2021) suggest that ploidy levels do not appear to be phylogenetically clustered. This suggests our results are unlikely to be simply due to relatedness and this could be further explored more robustly by incorporating a larger data set.

Under high-intensity fire, while ploidy may not affect the seed dormancy-breaking threshold, polyploid *Pomaderris* could perhaps emerge from deeper within the soil profile due to their larger seeds (Bond et al., 1999; Hanley et al., 2003; Liyanage and Ooi, 2017a). Taller and larger seedlings enhance the competitive ability of a plant, providing polyploid *Pomaderris* a higher potential to overgrow surrounding vegetation and better compete for light in a post-fire environment. The potential higher competitiveness of polyploid *Pomaderris* species aligns with previous studies that have found a positive correlation between seed mass and seedling height (Liancourt et al., 2009). Our study has demonstrated that polyploid *Pomaderris* may have higher potential competitiveness than diploids in fire-prone habitats. However, there are other stochastic factors that could affect seedling establishment in post-fire environments (Moles and Westoby, 2004a), and the interaction of ploidy with these factors needs further study in the field.

Conservation management has often focused solely on the adult plant persistence niche. However, the long-term recovery and persistence of a species is also dependent on the regeneration niche (Grubb, 1977; Jiménez-Alfaro et al., 2016; Saatkamp et al., 2019). Our study shows that seed and seedling traits exhibited during regeneration of narrow range species did not differ significantly from widespread species. However, ploidy was related to a large difference in overall regeneration performance of diploid and polyploid *Pomaderris*. Among the many threatened *Pomaderris* taxa, the regeneration niche of diploids should be considered as potentially limiting for populations because of their smaller seed size, slower germination rate and smaller seedlings. This insight to the regeneration phase may need to be considered when planning and prioritising management of threatened species.

## Data Availability Statement

The datasets presented in this study can be found online at Figshare at: https://doi.org/10.6084/m9.figshare.15042972.

## Author Contributions

MO and LG conceived the ideas. JC, MO, and LG designed methodology and led the writing of the manuscript. JC and MO collected the data and analysed the data. All authors contributed to the article and approved the submitted version.

## Funding

The project was supported by funding from the NSW Environmental Trust (grant number 2015/RD/0004). JC also received support from the UNSW Sonja & Huddle Award. A NSW Department of Planning, Industry and Environment Saving our Species Program grant provided funding to MO. MO is also supported by the NSW DPIE Bushfire Risk Management Research Hub. Funding for open access publication provided by CSIRO.

## Conflict of Interest

The authors declare that the research was conducted in the absence of any commercial or financial relationships that could be construed as a potential conflict of interest.

## Publisher’s Note

All claims expressed in this article are solely those of the authors and do not necessarily represent those of their affiliated organizations, or those of the publisher, the editors and the reviewers. Any product that may be evaluated in this article, or claim that may be made by its manufacturer, is not guaranteed or endorsed by the publisher.
