# Supplementary material for: Polyploidy but Not Range Size Is Associated With Seed and Seedling Traits That Affect Performance of Pomaderris Species
Source: Front Plant Sci. 2022 Jan 13;12:779651. doi: 10.3389/fpls.2021.779651 (PMC8793627; doi:10.3389/fpls.2021.779651)
Supplement: Supplementary file 1 [file Table_1.DOCX]

**Appendix S1**. Species available for each measured trait

| **Species** | **Ploidy** | **Range size** | **Seed**  **mass** | **T_50_** | **G_20_** | **Seed Mortality** | **RGR Stem length** | **Stem length** | **Biomass SRR** | **Biomass** | **Survival** |
| --- | --- | --- | --- | --- | --- | --- | --- | --- | --- | --- | --- |
| *P. adnata* | diploid | narrow range | √ | √ | √ | √ | √ | √ | √ | √ | √ |
| *P. andromedifolia* | polyploid | widespread | √ | √ | √ | √ | √ | √ | √ | √ | √ |
| *p. bodalla* | diploid | narrow range | √ | √ | √ | √ | √ | √ | √ | √ | √ |
| *P. brunnea* | diploid | narrow range | √ | √ | √ | √ | √ |  |  |  |  |
| *P. cotoneaster* | polyploid | narrow range | √ | √ | √ | √ | √ | √ | √ | √ | √ |
| *P. elachophylla* | polyploid | narrow range | √ |  |  |  |  |  |  |  |  |
| *P. eriocephala* | polyploid | widespread | √ | √ | √ | √ | √ | √ | √ | √ | √ |
| *P. intermedia* | polyploid | widespread | √ | √ | √ | √ | √ | √ | √ | √ | √ |
| *P. lanigera* | diploid | widespread | √ | √ | √ | √ |  |  |  |  |  |
| *P. ligustrina subsp. ligustrina* | diploid | widespread | √ | √ | √ | √ | √ | √ | √ | √ | √ |
| *P. pallida* | polyploid | narrow range | √ | √ | √ | √ | √ | √ |  |  | √ |
| *P. prunifolia* | polyploid | widespread | √ |  | √ | √ |  |  |  |  | √ |
| *P. reperta* | polyploid | narrow range | √ | √ | √ | √ | √ | √ | √ | √ | √ |
| *P. velutina* | diploid | widespread | √ | √ | √ | √ | √ | √ | √ | √ | √ |
| *P. walshii* | polyploid | narrow range | √ | √ |  |  |  |  |  |  |  |

**Appendix S2**. Mean soil weight (mg) and standard error over a 9-day duration of no watering.

Soil gravimetric water content (θ_g_) was calculated using the formula:

$$Gravimetric soil water content=\frac{wet weight-dry weight}{dried}weight$$

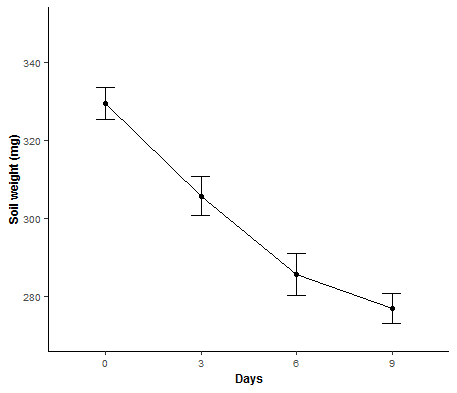


**Appendix S3**. Model selection statistics for various candidate models on seed traits. The term in brackets indicates that species was included as a random effect. AIC_c_ is the measure of model fit. Top models with delta < 2 which provide substantial support for each trait are listed. For G_20_, a model with at least one predictor was chosen as the optimal model despite a delta value slightly higher than 2.

| **Trait** | **Model structure** | **AIC_c_** | **Delta** |
| --- | --- | --- | --- |
| Seed mass | Seed mass ~ Ploidy + (1\|Species) | -167.0 | 0.00 |
| T_50_ | T_50_ ~ Ploidy + (1\|Species) | -293.4 | 0.00 |
| G_20_ | G_20_ ~ 1+ (1\|Species) | -49.7 | 0.00 |
| G_20_ | G_20_ ~ Ploidy + (1\|Species) | -47.8 | 1.88 |
| Seed mortality | Survived or Dead ~ Ploidy + (1\|Species) | 112.9 | 0.00 |

AIC_c_ = Akaike’s Information Criterion corrected for small sample sizes

**Appendix S4**. Model selection statistics for various candidate models on seedling traits. The term in brackets indicates that species was included as a random effect. AIC_c_ is the measure of model fit. Top models with delta < 2 which provide substantial support for each trait are also listed.

| **Trait** | **Model structure** | **AIC_c_** | **Delta** |
| --- | --- | --- | --- |
| RGR stem length | RGR of stem length ~ Range size + Treatment + (1\|Species) | -2730.8 | 0.00 |
| Stem length | Stem length ~ Ploidy level + (1\|Species) | 1738.9 | 0.00 |
| SRR | Shoot to root ratio (SRR) ~ Treatment + (1\|Species) | 100.4 | 0.00 |
| Biomass | Biomass ~ Ploidy level + Treatment + (1\|Species) | 426.7 | 0.00 |
| Survival | Survived or Dead ~ Treatment | 66.4 | 0.00 |

AIC_c_ = Akaike’s Information Criterion corrected for small sample sizes
